# A Study of 358 Cases of Locally Advanced Nasopharyngeal Carcinoma Receiving Intensity-Modulated Radiation Therapy: Improving the Seventh Edition of the American Joint Committee on Cancer T-Staging System

**DOI:** 10.1155/2017/1419676

**Published:** 2017-02-07

**Authors:** Qin Zhou, Yuxiang He, Yajie Zhao, Yin Wang, Weilu Kuang, Liangfang Shen

**Affiliations:** Department of Oncology, Xiangya Hospital, Central South University, No. 87, Xiangya Road, Changsha, Hunan Province 410008, China

## Abstract

To evaluate the rationality and limitations of the seventh edition of the American Joint Committee on Cancer (the 7th AJCC edition) T-staging system for locally advanced nasopharyngeal carcinoma (NPC). The prognosis of 358 patients with stage T3/T4 NPC treated with intensity-modulated radiotherapy (IMRT) was analyzed with the Kaplan–Meier method or the log-rank test. The 7th AJCC staging system of NPC has some limitations in that the T category is neither the significant factor in OS/LRFS nor the independent prognostic factor in OS/LRFS/DMFS/DFS (*P* > 0.05). After adjustment by anatomic structures, univariate analysis has shown that the adjusted-T category has statistical significance between T3 and T4 for OS (86.4% and 71.3%, *P* = 0.002), LRFS (97% and 90.9%, *P* = 0.048), DMFS (90.9% and 77.2%, *P* = 0.001), and DFS (86.2% and 67.5%, *P* = 0.000), and multivariate analysis has shown that the adjusted-T category is an independent prognostic factor for OS/DMFS/DFS (with the exception of LRFS). Then, GTV-P was taken into consideration. Multivariate analysis showed that these nT categories serve as suitable independent prognostic factors for OS/DMFS/DFS (*P* < 0.001) and LRFS (HR = 3.131; 95% CI, 1.090–8.990; *P* = 0.043). The 7th AJCC staging system has limitations and should be improved by including the modifications suggested, such as anatomic structures and tumor volume adjustment.

## 1. Introduction

Nasopharyngeal cancer (NPC) is among the most common malignant tumors in China, especially in the south. The incidence accounted for about 40% of the world's new cases according to the World Health Organization's GLOBOCAN reported data of 2012 [1]. Because of the complex anatomic location and high radiosensitivity of nonmetastatic NPC, radiotherapy (RT) is the recommended treatment. With progress in combined therapy and radiation therapy equipment, efficacy has significantly improved. However, there is still a relatively high local failure rate and distant metastases have occurred in patients with locally advanced NPC.

A reasonable clinical staging system is crucial for guiding treatment strategy and predicting prognosis. Such a system should have the following characteristics: consistency within the same risk group, heterogeneity among the risk groups, a balanced distribution of cases among the groups, and effective prediction of survival. At present the main treatment modality for NPC is still based on the TNM staging system. The breakthrough of the seventh edition of AJCC staging system (the 7th AJCC edition) involved the use of MRI—offering the advantage of higher resolution of soft tissue—as a basis for clinical staging [2].

Comprehensive treatment including intensity-modulated radiation therapy (IMRT) has become the standard treatment of NPC, as a result of which the traditional prognostic model based on anatomic structure has been challenged. Multivariate analysis of many studies including those from our department showed that, in the IMRT treatment model, T category is not an independent prognostic factor [3–6]. Another study [7] found that in LRFS there was no difference between T2 and T1 staging; they recommended that T2 be classified as T1. Furthermore, the study of 1241 NPC patients [8] showed that local control of skull involvement was similar to that of T1/T2 staging and that there was no significant difference in local control between patients in the T1 to T3 categories treated with IMRT. In addition, the difference in the T4 group was also obvious. Results from the studies [9, 10] demonstrated that the prognosis of T4 patients with involvement of a single masticator space is better than that of others in the same stage.

Although numerous studies have shown that the 7th AJCC edition is superior to past systems and that the N category still has very good predictive value [11, 12], the current TNM staging system also has limitations. For one thing, it is difficult to carry out individualized therapy under the current staging system. Prognoses vary widely within the same T stage, which is based only on the anatomic structures involved, especially for patients with locally advanced NPC. For another, the current staging system cannot distinguish the real risk factors influencing a patient's prognosis. Take tumor volume as an example. Many studies strongly suggest that the incorporation of GTV-P could further improve the accuracy of the current T category [13, 14], particularly in predicting local failure—a conclusion also reached by our previous studies.

Therefore it is an urgent matter to draft a more accurate staging system to better predict risk factors and guide both stratification and treatment in NPC. Our retrospective study analyzes the prognostic factors of 358 patients with locally advanced NPC who received IMRT as well as comprehensive treatment. It should serve to explore the limitations of the current situation as well as to suggest ways of improving the 7th AJCC edition.

## 2. Methods and Materials

### 2.1. Patient Characteristics

We performed a retrospective study of 358 consecutive patients with locally advanced NPC (T3/T4 N0–3M0) treated with IMRT between August 2008 and December 2011 at the Xiangya Hospital of Central South University (Changsha, Hunan Province, China) [15]. All cases underwent nasopharyngeal biopsy, non-contrast-enhanced and contrast-enhanced MRI of the nasopharyngeal and cervical region to assess locoregional involvement. In addition to CT/MRI examinations of the nasopharynx and neck, the pretreatment workup also included a complete medical history, physical examination, chest X-ray, and/or CT (all patients with N3 disease underwent CT of the chest), B-ultrasound scan of the abdomen and neck, bone scan, and routine laboratory analysis. The patients' clinical characteristics are summarized in Table 1. The study was approved by the ethics committee of the Xiangya Hospital (approval number 201111086).

### 2.2. Clinical Staging

All patients were investigated by physical examination, endoscopic examination, and imaging studies with both MRI and CT. The CT and MRI scans for each patient were independently reviewed by 2 senior clinicians from the Departments of Radiology and Oncology. Additional investigations for systemic workup were carried out, such as chest X-ray, abdominal ultrasound, bone scan, and positron emission tomography-CT (PET-CT) if necessary for patients with clinical features or biochemical abnormalities suspicious of distant metastases. All patients were staged according to the 7th AJCC staging system.

### 2.3. Tumor Volume Measurement and Grouping

The CT and MRI images were fused on the radiotherapy treatment planning system (TPS, Varian Medical System, Inc., Palo Alto, CA, USA) and the gross target volume of primary tumor of the nasopharynx (GTV-P) was manually delineated on the fused images. Since the retropharyngeal lymph nodes were indistinguishable from the primary tumor of NPC patients, the involved retropharyngeal lymph nodes were included as part of the GTV-P in our study. The target was independently reviewed by two senior clinicians in the Departments of Radiology and Oncology. In the event of controversy, the agreement was reached through the discussions of staff from radiotherapy department. The tumor volume was automatically calculated by the planning system. Based on the results of our previous study [16], we selected a cut-off value of 46.4 mL of tumor volume to regrouping.

### 2.4. Treatment and Follow-Up

All patients were treated with IMRT using 6-MV X-ray once a day 5 times a week. The specific target and dose constraints to critical normal structures were defined as described in our previous studies [16, 17]. The prescribed doses were 66–75.9 Gy for the PGTVnx, 69.96–72.6 Gy for PGTVnd, 59.4–64.0 Gy for PTV1, and 50.0–54.0 Gy for PTV2. The doses to the PTV2 were administered in 28 fractions and other doses in 33 fractions. If the GTV-P decreased obviously according to the repeat-MRI taken in 20 fractions or the fixed mask is not suitable, the second CT and planning must be redone. Dose limits for the target tissue and plan evaluation were based on the criteria of the Radiation Therapy Oncology Group 0225. Additional treatment with chemotherapy, such as neoadjuvant chemotherapy, adjuvant chemotherapy, and concurrent chemotherapy, was given to 337 patients with advanced disease. Various regimens of cytotoxic drugs (mostly cisplatin-based) were described in our previous study [16].

The median follow-up time was 58 months (range, 3–78 months), with 96.8% of patients finishing a complete 5-year follow-up. The follow-up methods included direct telephone calls to patients or hospital visits to the patients. After radiotherapy, follow-up examinations were conducted every 3 months in the first 2 years, once every 6 months in years 2 to 5, and annually thereafter. The criterion of tumor relapse, overall survival (OS), local relapse-free survival (LRFS), distant metastasis-free survival (DMFS), and disease-free survival (DFS) were defined as described in our previous study [16].

### 2.5. Statistical Analysis

All statistical analyses were performed using the Statistical Package for the Social Sciences version 17.0 (SPSS Inc., Chicago, IL, USA). Survival curves (OS, LRFS, DMFS, and DFS) were created with the Kaplan–Meier method and differences were compared with the log-rank test. Log-rank test was used for univariate analysis and multivariate analysis was performed using the Cox proportional hazards model. Receiver operating characteristic (ROC) curves were used to identify the cut-off values for different endpoints [16]. The areas under the curve (AUC) were used to assess the prognostic value of different T-staging system. The criterion for statistical significance was set at *α* = 0.05 and *P* values were based on two-sided tests.

## 3. Results

### 3.1. Patients' Clinical Characteristics

The clinical characteristics of the patients were presented in Table 1, which showed that age, gender, histological type, N staging, radiotherapy, chemotherapy, and other factors had no statistical significance for NPC. However, tumor volume of patients in the T4 category was significantly greater than that for patients classified in T3, which represented a significant statistical difference (*P* <0.001). In the distribution of our 358 locally advanced NPC cases, more were classified in the T4 than in the T3 category. The 7th AJCC edition was adopted for all patients.

### 3.2. The Limitations of the AJCC 7th T-Staging System

#### 3.2.1. Prognosis of Different T Categories

As defined by the 7th AJCC edition, there was statistical significance in N1 staging of 5-year OS and DFS between the T3 and T4 categories (Table 2); *P* values were 0.047 and 0.014. There were no significant differences between the other N categories, such as N0/N2/N3. To our surprise, the LRFS of patients at T3 in N2 staging is lower than that for the patients in T4 staging (81% versus 97.7%, *P* = 0.002). The 5-year OS/LRFS in all patients of T3 and T4 staging was not different (*P* > 0.05). After adjustment for age, gender, histological type, clinical stage (the 7th AJCC edition of T category and N category), tumor volume, chemotherapy, and radiotherapy dose, multivariate analysis found that the T category of AJCC 7th edition was not an independent prognostic factor (data in supplementary materials available online at https://doi.org/10.1155/2017/1419676). Therefore it does not make sense to classify locally advanced NPC treated with IMRT according to the current system.

#### 3.2.2. Prognosis of Different Tumor Volumes in the T4 Category

From clinical experience and our data [16], we found that patients in the same T category, especially T4 staging, had large discrepancy of tumor volumes. And these tumor volumes and T categories were often related to particular anatomic structures, such as the lateral pterygoid muscle (LPM) or brainstem (which is a dose-limited organ). Multivariate analyses of our previous study [16] showed that GTV-P was an independent prognostic factor of survival index (OS/LRFS/DMFS/DFS).

We therefore divided the T4 patients into 2 groups according to primary tumor volume [16], with a dividing line at 46.4 mL, determined by receiver operating characteristic (ROC) curves.  Table 3 shows the significant differences in 5-year OS/LRFS/DMFS/DFS. In fact, it shows that the prognoses of patients even in the same T4 stage are significantly different.

#### 3.2.3. Distribution of Anatomic Structures

Among 358 NPC patients staged by the 7th AJCC edition criteria, the rates of invasion into the medial pterygoid muscle (MPM) and skull were the highest, accounting for 269 (75.5%) and 284 (79.3%) patients, respectively. This was followed by invasion of the cavernous sinus, which was seen in 130 (36.3%) patients, and infiltration of the paranasal sinuses (PS) in 128 (35.8%) patients. However, orbital infringement was noted in only 4 patients and invasion of throat was seen in just 1 patient. Either the orbital or throat invasion usually occurred alongside erosion with other structures in the T4 category. In short, we took 6 anatomic sites but not the orbital or throat sites into consideration.

Table 4 lists the 6 anatomic sites involved by the primary tumor and the corresponding survival statistics. As the table shows, there were no significant differences in 5-year OS/LRFS/DMFS/DFS regardless of involvement of the MPM or cranial nerve, while patients with involvement of the PS had a relatively worse prognosis. From the 7th AJCC edition's multivariate analysis of survival (data in supplementary materials), we know that involvement of the PS is an independent risk factor in OS/LRFS/DMFS/DFS (*P* < 0.05) and should therefore be classified as stage T4. Because MPM is not an independent risk factor in predicting survival (*P* > 0.05), it should be classified as stage T3. Involvement of the cranial nerve is usually seen together with infiltration into other areas; hence it has no significant influence on staging.

### 3.3. Proposal for a New T-Staging System

#### 3.3.1. Adjustment of T3/T4 Staging of NPC by Anatomic Structure

**Figure 1:**
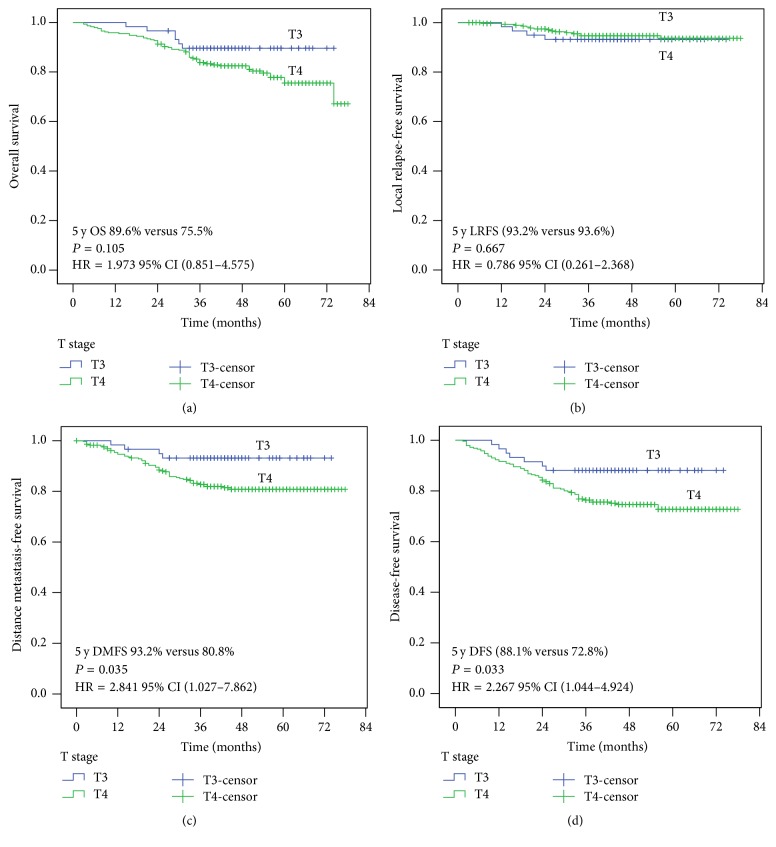
Survival analyses of NPC patients with T3/T4 NPC in the 7th AJCC T-staging system.

Figure 1 shows the Kaplan–Meier curves for the 5-year survival rate of patients staged with the 7th AJCC staging system. There was a significant difference between the groups of DMFS/DFS (*P* < 0.05). However, the differences in 5-year OS between T3 and T4 categories was not statistically significant (89.6% and 75.5%, *P* = 0.105), nor was LRFS (93.6% and 93.2%, *P* = 0.667).

According to the data already presented, we modified the staging of NPC with invasion of the MPM and skull base (including bone erosion) as T3, while involvement of the PS was staged as T4. That is, the adjusted T4 category includes the following structures: LPM, masticator space beyond the lateral pterygoid, intracranial, cavernous, and PS structures. The results of single-factor analysis indicate that the adjusted T-staging includes a remarkable difference with regard to 5-year OS/LRFS/DMFS/DFS (all *P* < 0.05) (Table 5). However, multivariate analysis still shows that the adjusted T-staging is an independent prognostic factor for OS/DMFS/DFS but not LRFS (HR = 1.290; 95% CI, 0.345–4.831; *P* = 0.705). Hence the adjusted T-staging system also had disadvantages and should be adjusted with other factors.

#### 3.3.2. Adjustment of the New T-Staging System by Primary Tumor Volume

**Figure 2:**
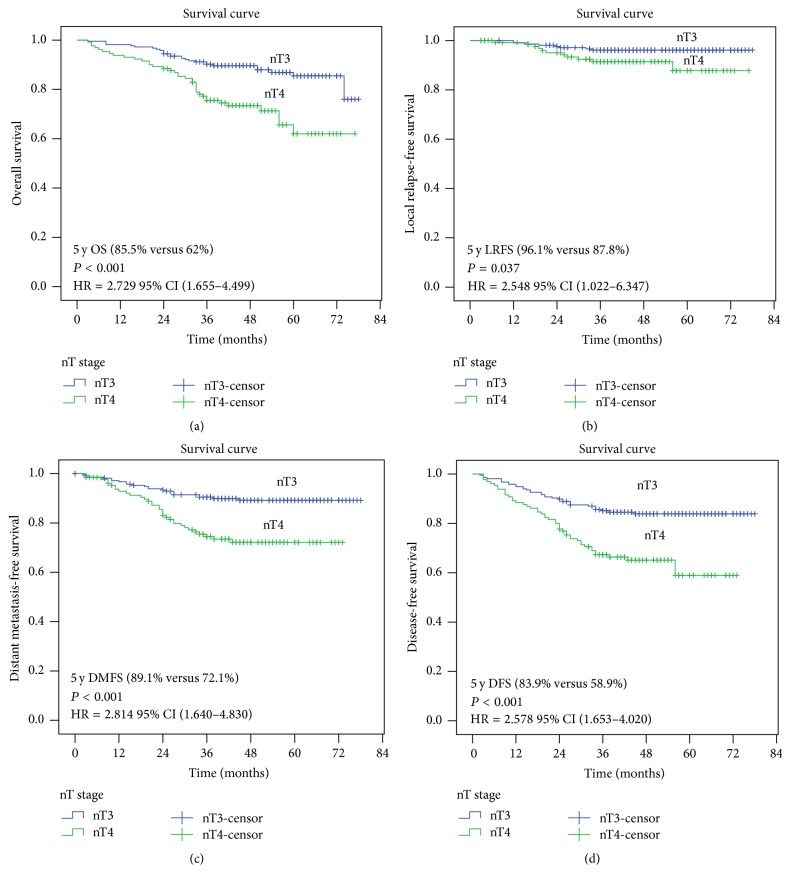
Survival analysis of NPC patients according to the new T-staging system (T4a + T3 = nT3; T4b = nT4).

From the foregoing data analysis, we know that the adjusted T-staging system is superior to the 7th AJCC edition in that it improves the OS/DMFS/DFS/LRFS classification of locally advanced NPC, but it still does not show an independent prognostic factor for LRFS.  Table 3 indicates that tumor volume is an important prognostic factor for locally advanced NPC. Taking this into consideration, we regrouped the adjusted T stages by including the consideration of tumor volume and then reanalyzed the survival prognosis.  Figure 2 shows that tumor volume is also a critical survival parameter for new T-staging, nT3 being equal to adjusted T3 + T4a (adjusted T4 ≤ 46.4 mL) and nT4 including only T4b (adjusted T4 > 46.4 mL). It is clear that nT-staging is a significant factor for 5-year OS/LRFS/DMFS/DFS (*P* < 0.05). And there was significant difference for LRFS in terms of the tumor volumes involved (the dividing line being set at 46.4 mL) for *P* = 0.037 (96.1% versus 87.8%).

Multivariate analysis (Table 6) shows that the new grouping (new T+ GTV-P) not only has better effect on predicting OS (HR = 2.504; 95% CI, 1.496–4.190; *P* < 0.001), DMFS (HR = 2.539; 95% CI, 1.463–4.404; *P* < 0.001), and DFS (HR = 2.365; 95% CI, 1.504–3.719; *P* < 0.001) but also has great predictive value for LRFS and is an independent risk factor for LRFS (HR = 3.131; 95% CI, 1.090–8.990; *P* = 0.043).

#### 3.3.3. Comparing the Prediction Efficacy of the Staging Systems

**Figure 3:**
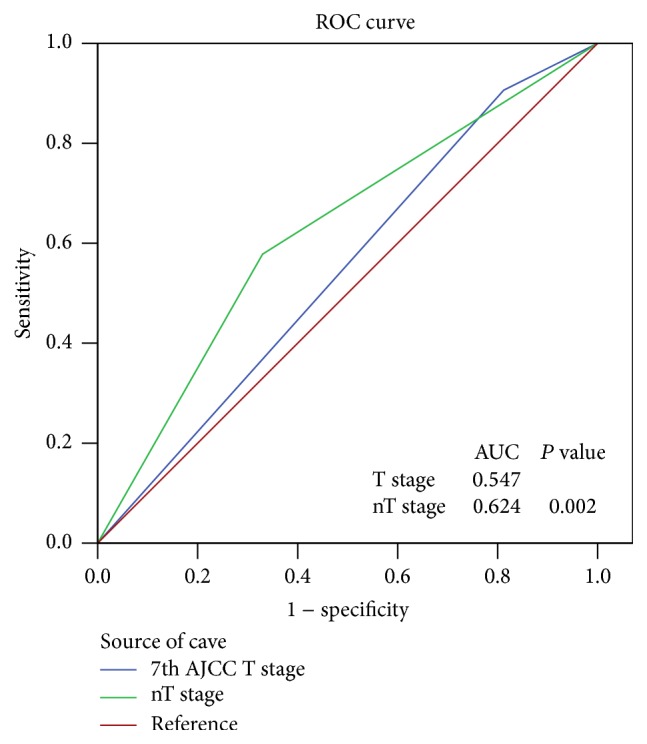
Receiver operator characteristic (ROC) curves for diagnosis in the two T-staging systems.

After tumor volume is taken into account in the adjusted T-staging system, it provides a more accurate prediction of survival for NPC patients. In addition, another analysis also confirms this result. We compared the survival prediction efficacy of the 7th AJCC edition and the new T-staging system, which includes the consideration of anatomic structures and GTV-P with the ROC curve.  Figure 3 shows that the new T-staging system had a significant statistical difference with the 7th AJCC edition (*P* = 0.002), and the area under the curve (AUC) between the 2 systems ranged from 0.54 to 0.624. These results indicate that the new T-staging system is superior to the 7th AJCC edition.

## 4. Discussion

Thus far the staging criteria used in the TNM system for NPC were based on anatomic structures alone [18]. There are some differences between the 7th AJCC edition and our new system. First, we classify involvement of the PS as T4 rather than as T3; second, we consider involvement of the MPM to be part of T3, whereas the 7th AJCC edition classifies it in T4. In our study, both the univariate and multivariate analyses showed that invasion of the PS had a significant effect on the outcomes of patients' OS/LRFS/DMFS/DFS. So, it was an independent prognostic factor and should be classified as T4. This result was opposite to that of Tian et al. [19] whose study showed that neither the maxillary, sphenoid, nor ethmoid was an independent prognostic factor in OS/DMFS/LRFS.

If a tumor did not extend to any other anatomic structures classified in the T3 or T4 category of the 7th AJCC edition but infiltrated only the MPM, the tumor volume was usually small and far from the brain stem or other OARS (organs at risk), so the ideal radiation dose can be delivered to the whole tumor. He et al. [15] reported that the dose coverage of tumor was seriously influenced by the distance between tumor and brain stem. MPM erosion seems to have little effect on prognosis; therefore, some studies [20, 21] showed that MPM erosion simply should be classified as T2. Our study confirmed the idea that MPM involvement was neither an independent risk factor nor an indicator of OS/LRFS/DMFS/DFS. As the patients in our study had only locally advanced NPC, it should be classified as T3 or even more earlier T-staging, which is consistent with the results reported elsewhere [22, 23].

Our data indicate that LPM involvement reduced OS and DMFS in our patients, although it was not an independent prognostic factor of OS/DMFS if other influential factors were adjusted in multivariate analysis. The result from univariate analysis may stem from several factors, one of which is that the LPM invasion often extended to other areas, such as the cavernous and paranasal sinuses. A study of 265 cases [24] found that involvement of either the pterygoid muscle or LPM alone has significant effect on prognosis, but more than 80% of these patients were treated with two-dimensional conformal radiotherapy (2D-CRT), not IMRT. In the 7th AJCC edition, invasion of the eyes and throat was part of the T4 category. However, very few of the patients in our study manifested such invasion. Therefore, for the sake of simplicity we did not take these structures into account in our system, which in this aspect is the same as 2008 T-staging system in China.

In our study, one of the significant factors affecting 5-year OS and DMFS was skull invasion; *P* values were 0.031 and 0.018, respectively. However, this was not an independent prognostic factor after adjusting for age and other factors in multivariate analysis (*P* > 0.05). In the 2D-CRT period, some researchers [25, 26] divided the patients into different subgroups to evaluate prognosis according to the extent of skull invasion or to classify extensive skull invasion as T4. In terms of current IMRT treatment modalities, the significance of skull involvement is still unknown. In our study, cranial nerve involvement had no significant effect on prognosis, which is consistent with the literature [27].

The results of our analysis suggest that it was reasonable to adapt the adjusted T-staging system to correspond with the 2008 T-staging system in China. After this was done, the distribution was better than that of the 7th AJCC edition. Moreover, the survival rate was more obviously different between adjusted T3 and T4 staging. In the multivariate analysis model of the 7th AJCC edition—taking age, gender, histological type, N stage, chemotherapy, and radiotherapy dose prescription into consideration—only age was an independent risk factor for LRFS. The T-staging of the 7th AJCC edition was not an independent prognostic factor of OS/LRFS/DMFS/DFS. Meanwhile the adjusted T-staging system is an independent prognostic factor for 5-year OS/DMFS/DFS but not the LRFS.

According to our analysis, the adjusted T-staging system provides a better prognostic value for OS/DMFS/DFS in patients with locally advanced NPC receiving IMRT. This may be mainly due to the regrouping of the MPM and PS. A retrospective analysis of 816 cases of nonmetastatic NPC [28] showed an opposite conclusion in that it was closer to the 7th AJCC edition. However, the patients were not confined to the locally advanced NPC group and the majority were receiving 2D-CRT.

Through the above analysis, we found that the adjusted T-staging system was superior to the 7th AJCC edition and whether it was satisfactory enough to guide treatment and evaluate prognosis was yet to be seen. In our study, we found that there was a significant difference for 5-year OS/DMFS/DFS in the adjusted T4-staging patients with different tumor volumes (≤46.4 mL and >46.4 mL). The data from the multivariate analysis adjusted by tumor volume showed that the adjusted T-staging was not an independent prognostic factor of OS (*P* > 0.05). This result showed tumor volume to be a more important factor for the prediction of prognosis. This observation was consistent with the literatures [5, 13, 29–31], where results showed tumor volume to be an independent prognostic factor whether treatment is 2D-CRT or IMRT. In the current IMRT era, T-staging according to anatomic structure only is not an accurate predictor of OS. We therefore resolved to take the tumor volume into account so as to optimize our new T-staging system.

Although the cases were distributed more reasonably in the adjusted T-staging of 358 patients, the tumor volume in adjusted T4-stage still had large difference. Taking tumor volume into consideration, especially the 201 patients in adjusted T4 category, we regrouped our patients according to tumor volume and analyzed their survival prognoses. In our previous study, the patients were divided into two groups, the threshold of tumor volume being 46.4 mL [16]. According to our statistical data, there was a significant difference in 5-year OS/DMFS/DFS but with no statistical significance for LRFS in adjusted T-staging. The prognosis of patients classified in adjusted T4-stage who had different tumor volumes varied. Thus we placed all the locally advanced NPC patients into two groups, one having a tumor volume of less than 46.4 mL (thus added to adjusted T3 staging, named nT3) and the second with a tumor volume larger than 46.4 mL (in adjusted T4-staging equal to nT4). Then the Kaplan–Meier method was used to estimate the survival curve. From Figure 2 we can see that the survival curves of OS/DMFS/DFS/LRFS between nT3 and nT4 were significantly different. Multivariate analysis shows that the nT-staging not only has a better effect on predicting OS/DMFS/DFS but also has a superior predictive effect for LRFS (HR = 3.131; 95% CI, 1.090–8.990; *P* = 0.043).

Then, using ROC curve analysis, optimum sensitivity, specificity, predictive values, and area under the ROC curve were evaluated. The areas under the curve of nT-staging and the 7th AJCC edition were 0.624 and 0.547, respectively (*P* = 0.002). The conclusion was that nT-staging has better prediction efficacy.

The 7th AJCC edition cannot effectively predict the outcome of OS/LRFS/DMFS/DFS and the adjusted system only based on anatomic structures cannot forecast the outcome of LRFS. When tumor volume is taken into account in determining T stage, this results in the superior prediction of all survival parameters. In the current IMRT era, it is possible to use the computer to estimate the volume of the primary tumor (GTV-P). Then it should be very easy to stage the locally advanced NPC on the basis of anatomic structures as well as tumor mass.

The current staging system for NPC is based on the anatomic structures involved. From our study and those of other researchers, several additional factors may influence the accuracy of clinical staging; these include tumor volume and the relationship between the tumor and the organs at risk. In addition, our study also has some limitations as its target population was only locally advanced NPC. Furthermore, many molecular markers—such as miRNA, EBV DNA—may also affect the prognostic. Because of the instability of current detection methods, there are no uniform standards, making the improvement of clinical staging a daunting task.

## Supplementary Material

Table1. After adjusted by some factors mentioned in the text, multivariate analysis found that the T category of the 7th AJCC edition was not an independent prognostic factor for OS (HR = 1.707, 95% CI, 0.732–3.981; *P* = 0.216), LRFS (HR = 1.341, 95% CI, 0.302–5.943, *P* = 0.700), DMFS (HR = 2.606, 95% CI, 0.940–7.225, *P* = 0.066) and DFS (HR = 2.041, 95% CI, 0.938–4.441 , *P* = 0.072). Table 2. Multivariate analysis of anatomical sites found that paranasal sinus involvement was an independent factor affecting OS (*P* =0.011), LRFS (*P* =0.011), DMFS and DFS (*P* <0.01). And cavernous sinus invasion was an independent factor affecting overall survival (HR = 1.886, 95% CI, 1.097–3.242, *P* = 0.022).

## Figures and Tables

**Table 1:** Characteristics of the 358 patients treated with IMRT.

Variable	AJCC7 T3 (*N* = 64)	T4 (*N* = 294)	*P* value
Age			
<50	46	186	0.191
≥50	18	108	
Gender			
Female	16	87	0.462
Male	48	207	
N stage			
N0	14	52	0.299
N1	20	93	
N2	24	94	
N3	6	55	
Histologic type			
Poorly differentiated SCC^*∗*^	58	275	0.407
Well-differentiated SCC^*∗*^	6	19	
Tumor volume			
≤46.4 mL	60	159	0.000
>46.4 mL	4	135	
Chemotherapy			
None	3	18	0.315
Concurrent or NACT^*∗*^ or adjuvant	14	34	
Concurrent + NACT	8	31	
Concurrent + adjuvant	1	9	
NACT + adjuvant	14	78	
NACT + concurrent + adjuvant	25	124	
Prescribed total dose			
<73.92 Gy	57	265	0.819
≥73.92 Gy	7	29	

^*∗*^SCC, squamous cell carcinoma; NACT, neoadjuvant chemotherapy.

**Table 2:** Univariate analysis of prognostic values of 386 patients staged at T3/T4.

AJCC 7th staging		5-year OS		5-year LRFS		5-year DMFS		5-year DFS	
		%	*P*	%	*P*	%	*P*	%	*P*
N0	T3 (12)	91.7	0.658	100	0.324	91.7	0.751	91.7	0.457
	T4 (51)	84.1		92		87.5		82	
N1	T3 (20)	100	0.047	100	0.183	100	0.062	100	0.014
	T4 (90)	75.2		89.2		83.5		71.5	
N2	T3 (21)	85.2	0.909	81	0.002	95	0.223	81	0.76
	T4 (92)	80.4		97.7		83.2		76.2	
N3	T3 (6)	62.5	0.817	100	0.634	66.7	0.916	66.7	0.821
	T4 (54)	59.7		95.9		65.9		55.6	
	ALL T3 (59)	89.6	0.105	93.6	0.667	93.2	0.035	88.1	0.033
	ALL T4 (287)	75.5		93.2		80.8		72.8	

OS, overall survival; LRFS, local relapse-free survival; DMFS, distant metastasis-free survival; DFS, disease-free survival.

**Table 3:** Patients staged at T4 with different primary tumor volumes.

AJCC 7th T4 staging (*n* = 287)	5-year OS		5-year LRFS		5-year DMFS		5-year DFS	
	%	*P*	%	*P*	%	*P*	%	*P*
Tumor volume								
≤46.4 mL (157)	84.6	0.000	97.2	0.01	87.8	0.001	82.4	0.000
>46.4 mL (130)	62		87.8		72.1		58.9	

**Table 4:** Univariate survival analysis of 6 anatomic sites.

Anatomy (number)	5-year OS		5-year LRFS		5-year DMFS		5-year DFS	
	%	*P*	%	*P*	%	*P*	%	*P*
MPM								
+(264)	83.1	0.150	93.6	0.812	80.8	0.078	73.2	0.156
−(82)	86.9		93.6		89.7		82.6	
Skull								
+(276)	74.7	0.021	93.8	0.220	80.9	0.092	71.9	0.018
−(70)	87.6		97		90.0		87.1	
PS								
+(125)	66.3	0.000	88.1	0.008	72.8	0.000	61.5	0.000
−(221)	84.1		96.6		88.6		83.4	
LPM								
+(100)	67	0.007	91.4	0.171	74.5	0.01	66.1	0.029
−(246)	81.6		94.5		86.4		78.9	
Cavernous								
+(126)	65.8	0.000	89.8	0.113	75	0.005	63.8	0.001
−(220)	84.8		95.6		87.4		82.2	
Cranial nerve								
+(67)	75.6	0.226	91.7	0.400	79.2	0.342	72.6	0.381
−(279)	77.4		93.8		83.8		75.8	

MPM, medial pterygoid muscle; PS, paranasal sinuses; LPM, lateral pterygoid muscle.

**Table 5:** Univariate analysis of prognostic factors in adjusted T-staging system adjusted by anatomic structure.

Adjusted T stage	5-year OS		5-year LRFS		5-year DMFS		5-year DFS	
	%	*P*	%	*P*	%	*P*	%	*P*
Adjusted T3 (145)	86.4	0.002	97	0.048	90.9	0.001	86.2	0.000
Adjusted T4 (201)	71.3		90.9		77.2		67.5	

**Table 6:** Multivariate analysis of the nT-staging system.

Variable	Regression coefficient	Standard error	*P* value	HR	95% CI	
					Low	Up
For OS						
Age (<50 versus ≥50 years)	.731	.258	.005	2.077	1.253	3.444
New T category (nT3 versus nT4)	.918	.263	.000	2.504	1.496	4.190
N category (N0 versus N1 versus N2 versus N3)	.391	.137	.004	1.479	1.131	1.934
Sex (male versus female)	−.164	.278	.556	.849	.493	1.463
Pathologic type (WHO I versus II-III)	.397	.473	.401	1.488	.589	3.757
Chemotherapy (yes versus no)	−.400	.483	.407	.670	.260	1.728
Radiation dose (<73.92 versus ≥73.92 Gy)	−.202	.437	.644	.817	.347	1.924
For LRFS						
Age (<50 versus ≥50 years)	1.759	.583	.003	5.806	1.853	18.197
New T category (nT3 versus nT4)	1.141	.538	.043	3.131	1.090	8.990
N category (N0 versus N1 versus N2 versus N3)	−.218	.270	.420	.804	.474	1.366
Sex (male versus female)	−.067	.587	.909	.935	.296	2.956
Pathologic type (WHO I versus II-III)	.462	.795	.561	1.587	.334	7.542
Chemotherapy (yes versus no)	.396	1.052	.707	1.486	.189	11.675
Radiation dose (<73.92 versus ≥73.92 Gy)	−.548	1.042	.599	.578	.075	4.456
For DMFS						
Age (<50 versus ≥50 years)	.262	.281	.351	1.300	.749	2.255
New T category (nT3 versus nT4)	.932	.281	.001	2.539	1.463	4.404
N category (N0 versus N1 versus N2 versus N3)	.432	.149	.004	1.540	1.151	2.061
Sex (male versus female)	−.144	.301	.633	.866	.480	1.564
Pathologic type (WHO I versus II-III)	.220	.524	.675	1.246	.446	3.480
Chemotherapy (yes versus no)	−.184	.612	.763	.832	.251	2.759
Radiation dose (<73.92 versus ≥73.92 Gy)	−.056	.439	.899	.946	.400	2.237
For DFS						
Age (<50 versus ≥50 years)	.510	.230	.026	1.666	1.062	2.613
New T category (nT3 versus nT4)	.861	.231	.000	2.365	1.504	3.719
N category (N0 versus N1 versus N2 versus N3)	.308	.120	.010	1.360	1.075	1.721
Sex (male versus female)	−.122	.250	.626	.885	.542	1.446
Pathological type (WHO I versus II-III)	.390	.400	.331	1.476	.674	3.236
Chemotherapy (yes versus no)	−.349	.439	.427	.705	.298	1.669
Radiation dose (<73.92 versus ≥73.92 Gy)	−.132	.378	.727	.876	.418	1.840

HR, hazard ratio; CI, confidence interval.

## Abbreviations

AJCC 7th edition: Seventh edition of the American Joint Committee on Cancer
NPC: Nasopharyngeal carcinoma
CT: Computed tomography
MRI: Magnetic resonance imaging
OS: Overall survival
LRFS: Local relapse-free survival
DFS: Disease-free survival
DMFS: Distant metastasis-free survival
IMRT: Intensity-modulated radiation therapy
2D-CRT: Second dimension-conformal radiation therapy
GTVnx: Gross target volume nasopharynx
GTVnd: Gross target volume positive lymph nodes
PTV: Planning target volume
GTV-P: Primary gross tumor volume
LPM: Lateral pterygoid muscle
MPM: Medial pterygoid muscle
PS: Paranasal sinuses
HR: Hazard ratio
CI: Confidence interval
TNM: Tumor nodes metastasis.

## Additional Points

*Availability of Data and Materials.* See Supplementary Material.
