# Supplementary material for: A Study of 358 Cases of Locally Advanced Nasopharyngeal Carcinoma Receiving Intensity-Modulated Radiation Therapy: Improving the Seventh Edition of the American Joint Committee on Cancer T-Staging System
Source: Biomed Res Int. 2017 Feb 7;2017:1419676. doi: 10.1155/2017/1419676 (PMC5318629; doi:10.1155/2017/1419676)
Supplement: Supplementary file 1 — Table1. After adjusted by some factors mentioned in the text, multivariate analysis found that the T category of the 7th AJCC edition was not an independent prognostic factor for OS (HR = 1.707, 95% CI, 0.732–3.981; P = 0.216), LRFS (HR = 1.341, 95% CI, 0.302–5.943, P = 0.700), DMFS (HR = 2.606, 95% CI, 0.940–7.225, P = 0.066) and DFS (HR = 2.041, 95% CI, 0.938–4.441 , P = 0.072). Table 2. Multivariate analysis of anatomical sites found that paranasal sinus involvement was an independent factor affecting OS (P =0.011), LRFS (P =0.011), DMFS and DFS (P <0.01). And cavernous sinus invasion was an independent factor affecting overall survival (HR = 1.886, 95% CI, 1.097–3.242, P = 0.022). [file 1419676.f1.docx]

Table 1. The multivariate models of influence factors on the prognosis of the 7th AJCC stage system

| End point | Variable | | Regression  coefficient | Standard  error | *P*-  value | HR | 95% CI | |
| --- | --- | --- | --- | --- | --- | --- | --- | --- |
|  |  |  |  |  |  |  | Lower | Upper |
| OS | | Age | .840 | .251 | .001 | 2.317 | 1.416 | 3.792 |
|  |  | N-AJCC | .432 | .131 | .001 | 1.540 | 1.190 | 1.992 |
|  |  | T-AJCC | .535 | .432 | .216 | 1.707 | .732 | 3.981 |
| LRFS | | Age | 1.800 | .578 | .002 | 6.047 | 1.950 | 18.757 |
|  |  | T-AJCC | .293 | .760 | .700 | 1.341 | .302 | 5.943 |
| DMFS | | N-AJCC | .451 | .145 | .002 | 1.570 | 1.182 | 2.085 |
|  |  | T-AJCC | .958 | .520 | .066 | 2.606 | .940 | 7.225 |
| DFS | | Age | .572 | .226 | .011 | 1.773 | 1.139 | 2.759 |
|  |  | N-AJCC | .330 | .117 | .005 | 1.391 | 1.106 | 1.749 |
|  |  | T-AJCC | .713 | .397 | .072 | 2.041 | .938 | 4.441 |

Table 2 Multivariate analysis of different anatomical sites invasion on the prognosis

| End point | Variable | Regression  coefficient | Standard  error | *P*-value | HR | 95% CI | |
| --- | --- | --- | --- | --- | --- | --- | --- |
|  |  |  |  |  |  | Lower | Upper |
| OS | Age | 0.781 | 0.252 | 0.002 | 2.184 | 1.334 | 3.576 |
|  | N-stage | 0.379 | 0.106 | 0.000 | 1.460 | 1.186 | 1.797 |
|  | CS | 0.634 | 0.276 | 0.022 | 1.886 | 1.097 | 3.242 |
|  | PS | 0.700 | 0.275 | 0.011 | 2.013 | 1.174 | 3.453 |
| LRFS | Age | 1.750 | .578 | .002 | 5.753 | 1.854 | 17.855 |
|  | PS | 1.368 | 0.540 | 0.011 | 3.928 | 1.364 | 11.313 |
| DMFS | N-stage | .398 | .117 | .001 | 1.488 | 1.183 | 1.872 |
|  | PS | 1.157 | 0.276 | 0.000 | 3.182 | 1.852 | 5.467 |
| DFS | Age | .525 | .225 | .020 | 1.690 | .020 | 1.690 |
|  | N-stage | .315 | .091 | .001 | 1.370 | .001 | 1.370 |
|  | PS | 0.925 | 0.233 | 0.000 | 2.521 | 1.598 | 3.576 |

PS:paranasal sinus CS: cavernous sinus
